# Linear energy transfer correction using Al₂O₃:Cr thermoluminescent and radiophotoluminescence glass dosimeters for therapeutic proton dosimetry

**DOI:** 10.1007/s12194-025-00942-x

**Published:** 2025-07-18

**Authors:** Weishan Chang, Hina Suzuki, Kenji Hotta, Puspen Chakraborty, Yusuke Koba, Nozomi Ohba, Kiyomitsu Shinsho

**Affiliations:** 1 Tokyo Metropolitan University, 7-2-10 Higashi-Ogu, Arakawa-Ku, Tokyo 116-8551 Japan; 2 National Cancer Center Hospital East, 1 6-5-1 Kashiwanoha, Kashiwa-Shi, Chiba 277-8577 Japan; 3 National Institutes for Quantum Science and Technology, 4-9-1 Anagawa, Inage-Ku, Chiba 263-8555 Japan; 4 Kawasaki Saiwai Hospital, 31-27 Omiyacho, Saiwai-Ku, Kawasaki-Shi, Kanagawa 212-0014 Japan

**Keywords:** Proton therapy, Audit dosimetry, TLD, RPLD, LET dependence, Luminescence

## Abstract

Solid-state luminescence dosimeters face challenge in achieving accurate dosimetry in proton therapy owing to the linear energy transfer (LET)-dependent response. In this study, we proposed a two-dosimeter-based methodology to improve the accuracy of proton dosimetry by correcting the LET-dependent response of a radiophotoluminescence glass dosimeter (RPLD) and an Al_2_O_3_:Cr-based ceramic-type thermoluminescence dosimeter (TLD) for postal dosimetry. The LET dependent response for the RPLD and Al_2_O_3_:Cr TLD was investigated using an unmodulated 235 MeV proton beam delivered by a passive scattering system. Both dosimeters were individually calibrated in terms of the absorbed dose to water using a 6 MV X-ray beam. The luminescence efficiency ratio between the RPLD and Al_2_O_3_:Cr TLD (${\eta }_{{\text{RPLD}, \, \text{Al}}_{2}{\text{O}}_{3}:\text{Cr}}$) was used as an index to determine the LET dependence correction factor for the RPLD and Al_2_O_3_:Cr TLD (${k}_{\text{LET}}^{\text{RPLD}}$ and ${k}_{\text{LET}}^{{\text{Al}}_{2}{\text{O}}_{3}:\text{Cr}}$). Modulated proton beams with different spread-out Bragg peak (SOBP) widths were used to evaluate the feasibility of the proposed two-dosimeter methodology. ${\eta }_{{\text{RPLD}, \, \text{Al}}_{2}{\text{O}}_{3}:\text{Cr}}$ decreased with increasing LET. ${k}_{\text{LET}}^{\text{RPLD}}$ and ${k}_{\text{LET}}^{{\text{Al}}_{2}{\text{O}}_{3}:\text{Cr}}$ were fitted using exponential curves. Proton dosimetry based on the proposed methodology underestimated the absorbed dose to water by an averages of 1.88% and 3.21% for RPLD and Al_2_O_3_:Cr TLD, respectively. This demonstrated the feasibility of the proposed methodology. Although the method shows promise for LET correction, the uncertainties in the LET-dependent correction factors, namely 2.39% for the RPLD and 5.84% for the Al₂O₃:Cr TLD, indicate the need for further refinement.

## Introduction

The energy transferred to the tissue by protons is inversely proportional to their velocity, resulting in maximum dose deposition at a certain depth in the tissue, known as the Bragg peak [1]. This property is favorable when considering the trade-off between administering the prescribed target dose and the dose to the surrounding normal tissue in radiotherapy [2]. Nevertheless, accurate dosimetry remains a critical challenge owing to variations in the linear energy transfer (LET) along the proton beam path. LET-dependent effects have been reported in many commercially available solid-state luminescence dosimeters (SSLDs) such as thermoluminescence dosimeters (TLDs) and radiophotoluminescence glass dosimeters (RPLDs). These SSLDs are widely used for in vivo dosimetry and postal dosimetry programs for interinstitutional dose intercomparisons [3–6].

A commercially used RPLD glass dosimeter of the GD-300 series (Asahi Techno, Japan), has been reported to exhibit an under-response of approximately 45% at an LET of 9.5 keV µm^−1^ [7–10]. In radiotherapy, luminescence efficiency (*η*) for SSLD is defined as the dose ratio between using SSLD and ionization chamber (IC). Chang et al. attempted to address the LET-dependent response issue by proposing a residual range (*R*_res_)-based method to correct the stopping power and the LET dependent effect of the *η* on the RPLD (${\eta }_{\text{RPLD}}$) [8]. Although the *R*_res_-based correction method exhibits limitations in the high-LET region, it is useful in the plateau region. Yasui et al. proposed an alternative correction method based on the calculated LET [9]. The LET-based correction method improved the accuracy of RPLD measurements to within 5% of the corresponding dose by an IC. However, its practical application is limited as it requires detailed nozzle information, which is often unavailable.

A Cr-doped Al_2_O_3_-based ceramic TLD (Al_2_O_3_:Cr TLD, A10, Ceramic Mfg. Co) was proposed for application as a postal dose audit dosimeter and quality assurance tool in photon radiotherapy owing to its high solidity and durability [11, 12]. Koba et al. investigated the LET dependence of *η* on Al_2_O_3_:Cr TLD (${\eta }_{{\text{Al}}_{2}{\text{O}}_{3}:\text{Cr}}$) using different species of charged particles [13]. They found that, in contrast to ${\eta }_{\text{RPLD}}$, which decreases with increasing LET, ${\eta }_{{\text{Al}}_{2}{\text{O}}_{3}:\text{Cr}}$ reached its maximum of 1.2 when irradiated with a 290 MeV carbon-ion (LET: 13.5 keV µm^−1^) and then decreased to 0.6 when the LET was 200 keV µm^−1^.

In proton therapy, the LET values are typically around 0.5–12 keV µm^−1^ where ${\eta }_{{\text{Al}}_{2}{\text{O}}_{3}:\text{Cr}}$ and ${\eta }_{\text{RPLD}}$ increases and decreases with increasing LET, respectively. This suggests an increase in the difference between ${\eta }_{{\text{Al}}_{2}{\text{O}}_{3}:\text{Cr}}$ and ${\eta }_{\text{RPLD}}$ with increasing LET. By taking the ratio of ${\eta }_{{\text{Al}}_{2}{\text{O}}_{3}:\text{Cr}}$ to ${\eta }_{\text{RPLD}}$, the denominator of dose by the IC can be eliminated and LET can be expressed as a function of the ratio between dose by the RPLD and the Al_2_O_3_:Cr TLD. Subsequently, the LET-dependent correction factors for both the RPLD and the Al_2_O_3_:Cr TLD can be obtained. This indicates the potential of the combined dosimetric approach. However, previously, only ${\eta }_{{\text{Al}}_{2}{\text{O}}_{3}:\text{Cr}}$ at limited LET values of 0.5, 2, and 13 keV µm^−1^ were investigated, leaving a significant gap in fully understanding the LET dependence of ${\eta }_{{\text{Al}}_{2}{\text{O}}_{3}:\text{Cr}}$.

In this study, we developed a two-dosimeter-based methodology to improve the accuracy of proton dosimetry by addressing the LET-dependent response of the RPLD and the Al_2_O_3_:Cr TLD for postal dosimetry. As the ${\eta }_{{\text{Al}}_{2}{\text{O}}_{3}:\text{Cr}}$ has not been comprehensively investigated in previous studies, we experimentally investigated their LET-dependent response using an unmodulated 235 MeV proton beam that was delivered by a passive beam delivery system. The proposed methodology was the experimentally validated using a modulated proton beam with different spread-out Bragg peak (SOBP) widths.

## Materials and methods

### Dose determination

The absorbed dose to water (*D*_w_) at beam quality *Q* using an SSLD (RPLD or Al_2_O_3_:Cr TLD) was estimated using the following equation:

1 $${D}_{w}^{\text{SSLD}}={M}_{Q}^{\text{SSLD}}\times {N}_{D,w}^{\text{SSLD}}\times {k}_{Q,{Q}_{0}}^{\text{SSLD}}\times {k}_{\text{LET}}^{\text{SSLD}},$$

where ${M}_{Q}^{\text{SSLD}}$ denotes the reading (rdg) of the SSLD corrected for the fading effect, ${N}_{D,w}^{\text{SSLD}}$ is the calibration coefficient (Gy rdg^−1^) in terms of *D*_w_ for the SSLD at the reference beam quality ${Q}_{0}$, ${k}_{Q,\text{Q}_{0}}^{\text{SSLD}}$ is the beam quality correction factor for the SSLD, and ${k}_{\text{LET}}^{\text{SSLD}}$ is an LET-dependent correction factor determined using the proposed two-dosimeter-based methodology. The fading effect correction factor *f* for Al_2_O_3_:Cr TLD varied with the elapsed time *t* [h] and was calculated as [11]

2 $$f\left(t\right)=-0.36169\times {\text{log}}_{10}\left(t\right)+1.7234.$$

For RPLD, *f* was set to 1 because all the RPLD was readout within one week after irradiation, and the fading effect was negligible (< 2% for 120 days) [5, 14].

${N}_{D,w}^{\text{SSLD}}$ is the ratio of ${D}_{w, {Q}_{0}}^{\text{IC}}$ and *D*_w_ determined using an IC at the reference beam ${Q}_{0}$, and ${M}_{{Q}_{0}}^{\text{SSLD}}$. In this study, ${N}_{D,w}^{\text{SSLD}}$ was determined individually for each SSLD to improve the accuracy of the measurements. Since the absorbed dose standard for proton beams has not been established, a 6 MV X-ray from LINAC (Elekta VersaHD) was used as the reference beam in this study.

${k}_{Q,{Q}_{0}}^{\text{SSLD}}$ for RPLD was calculated using Eq. (3), as reported in the previous study [8]:

3 $${k}_{Q,{Q}_{0}}^{\text{RPLD}}\left({R}_{\text{res}}\right)=0.994+0.052{e}^{-0.537{R}_{\text{res}}}.$$

Similarly, the beam quality correction factor for the Al_2_O_3_:Cr TLD, ${k}_{Q,{Q}_{0}}^{{\text{Al}}_{2}{\text{O}}_{3}:\text{Cr}}$ was derived using the stopping power ratio between Al_2_O_3_:Cr and water, based on the PSTAR program [15], as follows:

4 $${k}_{Q,{Q}_{0}}^{{\text{Al}}_{2}{\text{O}}_{3}:\text{Cr}}\left({R}_{\text{res}}\right)=1.0165+0.0347{e}^{-0.522{R}_{\text{res}}}.$$

### LET-dependent response correction factor ${k}_{\text{LET}}^{\text{SSLD}}$

The following framework indicates a method for using the distinct LET-dependent response between the RPLD and Al_2_O_3_:Cr TLD for LET estimation in unknown radiation fields. After accessing the relationship between LET and $\eta$ for SSLD, i.e., the ${\eta }_{\text{RPLD}}(\text{LET})$ and ${\eta }_{{\text{Al}}_{2}{\text{O}}_{3}:\text{Cr}}(\text{LET})$, the relationship between the ratio of ${\eta }_{\text{RPLD}}$ and ${\eta }_{{\text{Al}}_{2}{\text{O}}_{3}:\text{Cr}}$ (${\eta }_{\text{RPLD},{\text{Al}}_{2}{\text{O}}_{3}:\text{Cr}}$) and LET was also derived as${\eta }_{\text{RPLD},{\text{Al}}_{2}{\text{O}}_{3}:\text{Cr}}(\text{LET})$. As ${\eta }_{\text{SSLD}}={M}_{Q}^{\text{SSLD}}\times {N}_{D,w}^{\text{SSLD}}\times {k}_{Q,{Q}_{0}}^{\text{SSLD}}/{D}_{w, Q}^{\text{IC}}$, ${\eta }_{\text{RPLD},{\text{Al}}_{2}{\text{O}}_{3}:\text{Cr}}(\text{LET})$ can be expressed as

5 $${\eta }_{\text{RPLD},{\text{Al}}_{2}{\text{O}}_{3}:\text{Cr}}(\text{LET})=\frac{{M}_\text{Q}^{\text{RPLD}}\times {N}_{D,w}^{\text{RPLD}}\times {k}_{\text{Q},\text{Q}_{0}}^{\text{RPLD}}/{D}_{w, \text{Q}}^{\text{IC}}}{{M}_\text{Q}^{{\text{Al}}_{2}{\text{O}}_{3}:\text{Cr}}\times {N}_{D,w}^{{\text{Al}}_{2}{\text{O}}_{3}:\text{Cr}}\times {k}_{\text{Q},\text{Q}_{0}}^{{\text{Al}}_{2}{\text{O}}_{3}:\text{Cr}}/{D}_{w, \text{Q}}^{\text{IC}}}$$

The LET value for a given measurement was determined by inverting the function. Given the ${k}_{\text{LET}}^{\text{SSLD}}$ is determined as the reciprocal of the ${\eta }_{\text{SSLD}}$, it can be expressed as:

6 $${k}_{\text{LET}}^{\text{SSLD}}=1/{\eta }_{\text{RPLD},{\text{Al}}_{2}{\text{O}}_{3}:\text{Cr}}(\text{LET})$$

### Dosimetry systems

#### RPLD dosimetry system

In this study, the RPLD dosimetry system pertaining to GD-302M (Asahi Techno Glass) and an automatic reader pertaining to FGD-1000 (Asahi Techno Glass) were used. The GD-302M is a cylinder with a diameter and height of 1.5 mm and 12 mm, respectively. The annealing condition and readout procedures were identical to those used in our previous study [8]. To improve accuracy, the timeline of the readout procedure—preheating and reading within one week after preheating—was consistent for each experiment in the present study.

#### Al_2_O_3_:Cr TLD dosimetry system

**Fig. 1:**
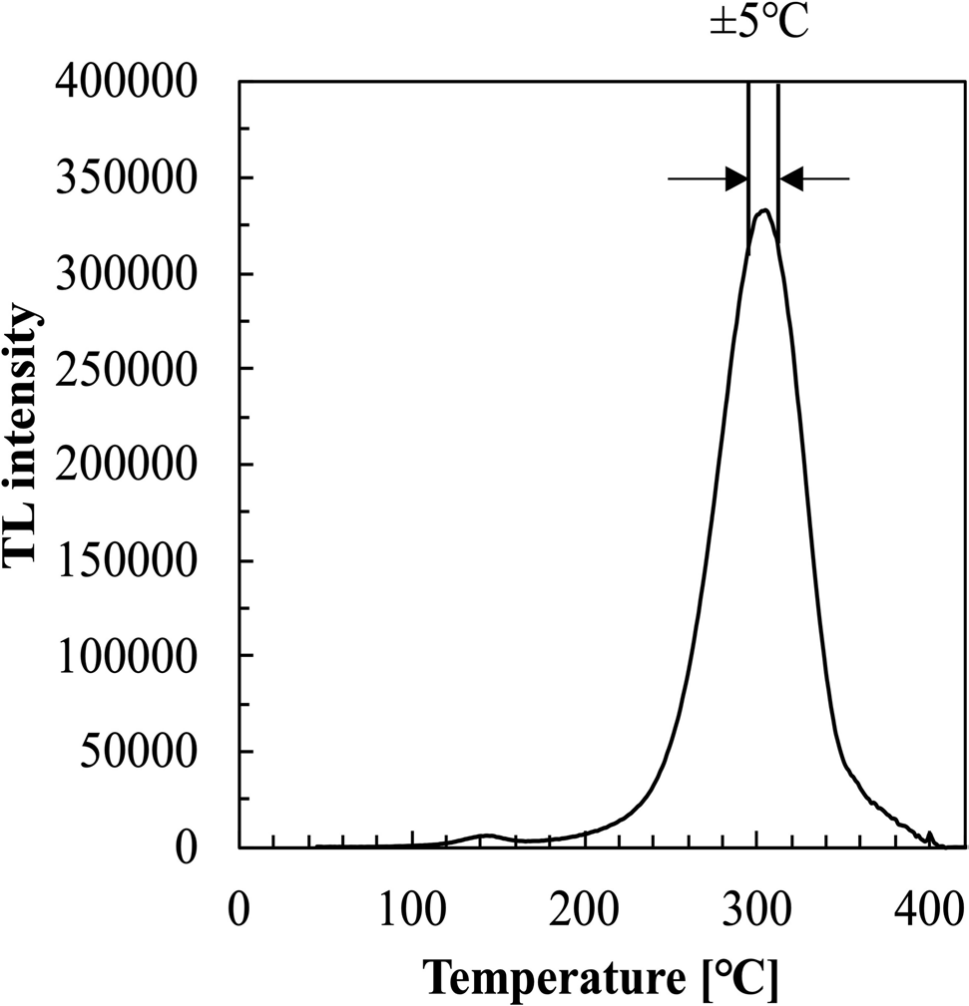
Glow curve for Al_2_O_3_:Cr TLD

The weight composition of the Al_2_O_3_:Cr TLD (A10, Chiba Ceramic Mfg. Co) is 99.5 wt% Al_2_O_3_ and 0.05 wt% Cr_2_O_3_. The dimensions of the Al_2_O_3_:Cr TLD used in this study was 10 mm × 10 mm × 0.7 mm [13]. The Al_2_O_3_:Cr TLDs were readout using an in-house developed glow curve readout system that contained a photon-counting unit (H11890-110, HAMAMATSU) with a collective lens, and programmable heat controller (SCRSHQA, SAKA-GUCHI). The detailed scheme of this readout system can be found at previous study [16]. Figure 1 shows the glow curve for Al_2_O_3_:Cr TLD that was heated from room temperature to 400 °C at a heating rate of 0.2 °C s^−1^. In this study, the TL between 40 °C and 400 °C was recorded to plot the glow curve. The main glow peak was observed at 310 °C, and the cumulated TL intensity between 305 °C and 315 °C was used to determine the absorbed dose rather than the total integral TL. This is because the total integral TL may contain the thermal radiation from the brass heating plate used in the TLD readout system. During heating, the brass plate emits infrared radiation that can be detected by the photomultiplier tube and consequently overestimate the TL signal. Al_2_O_3_:Cr TLDs were enveloped in a dark box immediately after irradiation to prevent light-induced decay and all TLDs were maintained at room temperature before readout.

### Experiment

#### LET dependence of luminescence efficiency

**Fig. 2:**
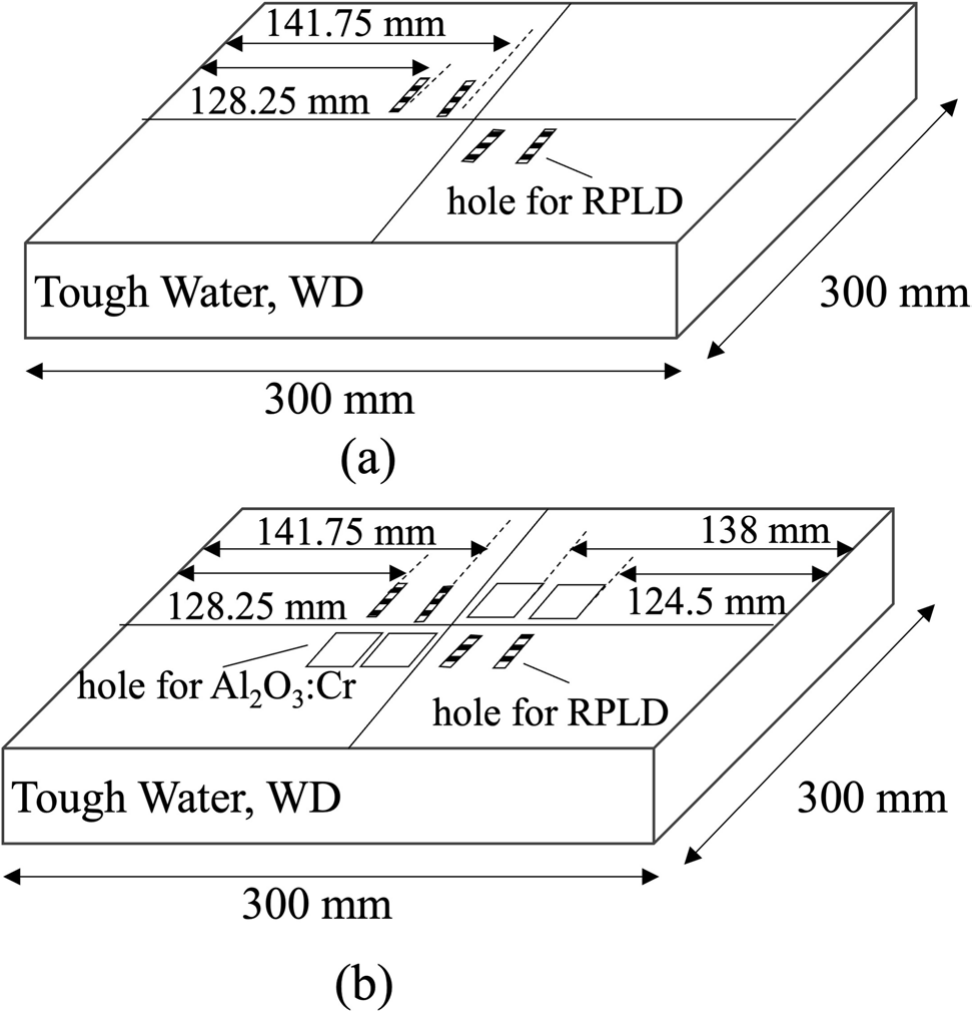
Customized tough water WD phantom used in the experiment: **a** phantom placed above the detectors, showing embedded positions for RPLDs only because the active region of the RPLD is located at its center; **b** phantom placed below the detectors, showing the embedded positions of both RPLDs and Al₂O₃:Cr TLDs

A non-modulated 235 MeV proton beams from the National Cancer Center EAST Hospital (NCCEH) was used to access the LET dependent luminescence efficiency of RPLD and Al_2_O_3_:Cr TLD. The Beam delivery system in the NCCEH was implemented with a double-scattering method to produce a uniform lateral dose distribution [17]. A water equivalent solid phantom, tough water WD (Kyoto Kagaku Co) was customized simultaneously hold the RPLD and Al_2_O_3_:Cr TLD for irradiation (Fig. 2). The geometric centers of the RPLD and front surfaces of the Al_2_O_3_:Cr TLD were set at the isocenter. Since the RPLD has a small diameter of 1.2 mm and an active volume diameter of 1 mm, aligning based on water-equivalent thickness would require impractically precise adjustments. Therefore, alignment was performed based on physical depth rather than water-equivalent thickness [18]. 1 Gy was delivered to the RPLDs and the Al_2_O_3_:Cr TLDs with 10 cm × 10 cm field. Four RPLDs and Al_2_O_3_:Cr TLDs were used for one irradiation condition.

**Fig. 3:**
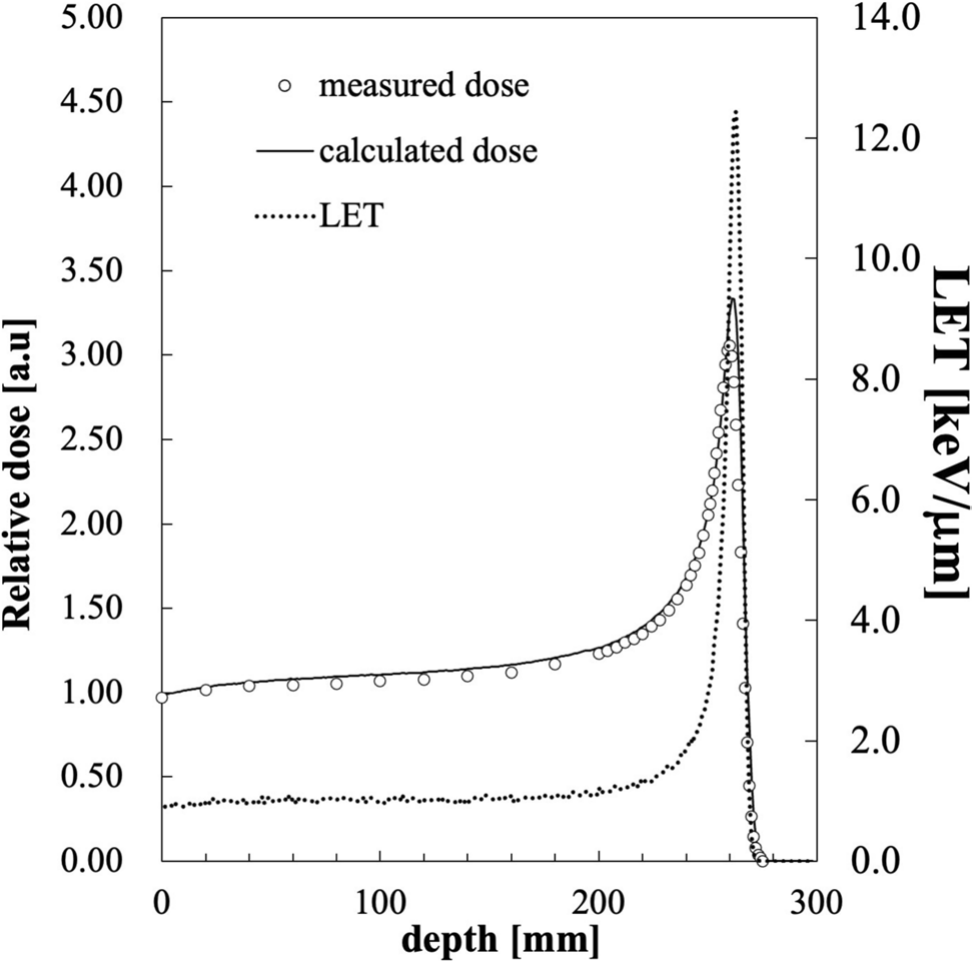
The relationship between measurement depths, LET, and dose distributions by measurement and MC calculation

The LET was varied using slabs made of poly(methyl methacrylate) (PMMA). In this study, the dose-averaged LET*,* which is a better parameter for estimating the relative efficiency of a detector in a proton beam when performing depth dose measurements [19], was calculated using the Monte Carlo simulation code PHITS (Particle and Heavy Ion Transport code system), version 3.26 [20]. Simulations were performed by assuming a Gaussian shape for the energy spectrum with mean energy of 226 MeV and the full width at half maximum of the Gaussian distribution was set as 0.6 MeV. A total of 2.5 × 10^8^ initial proton particles were set for simulation and transport parameter such as the energy and angular straggling for protons followed the recommendation as shown in Table 1. All simulations were conducted on 16 cores of the Intel Xeon Gold running at 2.9 GHz and the depth dose distribution was calculated with statistic uncertainties within 0.2%. Figure  3 shows the relationship between measurements depths, LET, and the dose distribution by measurement and MC calculation. The LET corresponding to the thickness of PMMA are listed in Table 2.

**Table 1:** Simulation related parameters used in this study

Parameter	Value	Explanation
nedisp	1	Energy straggling option for charged particle and nuclei; 1 is set to refer Landau Vavilov energy straggling
nspred	2	Options for Coulomb diffusion (angle straggling); 2 is set to have Coulomb diffusion by the Lynch’s formula based on the Moliere theory
ascat1	13.6	Parameter A in the Lynch’s formula for nspred = 2
ascat2	0.038	Parameter B in the Lynch’s formula for nspred = 2
Negs	1	Parameter related to electron, positron, and photon transport; 1 is to transport electrons, positrons, and photons based on the EGS5 algorithm
Epstfl	1	Parameter related to density correction factors; 1 is set to use ICRU90 density correction factors

**Table 2:** PMMA thickness vs. LET in an unmodulated 235 MeV proton beam

PMMA thickness [mm]	95	105	154	206	217	234	238	241	243	244
LET [keV µm^−1^]	0.89	1.05	1.19	1.43	1.55	1.87	2.01	2.15	2.27	2.35
PMMA thickness [mm]	245	246	247	248	249	251	252	253	254	255
LET [keV µm^−1^]	2.43	2.52	2.63	2.76	2.91	3.28	3.49	3.67	3.8	3.93

#### Verification experiment

Modulated (width of SOBP = 4, and 10 cm) 235 MeV proton beams from the NCCEH were used to validate the feasibility of the developed two-dosimeter-based methodology. Doses in the plateau region (5 cm) and SOBP region (center of the SOBP, 1 cm proximal and 1 cm distal to the center of SOBP) were measured for comparison. The irradiation dose was 1 Gy determined using an Advanced Markus IC (Type 34045 PTW, Germany) at each depth.

## Results

### LET dependence

**Fig. 4:**
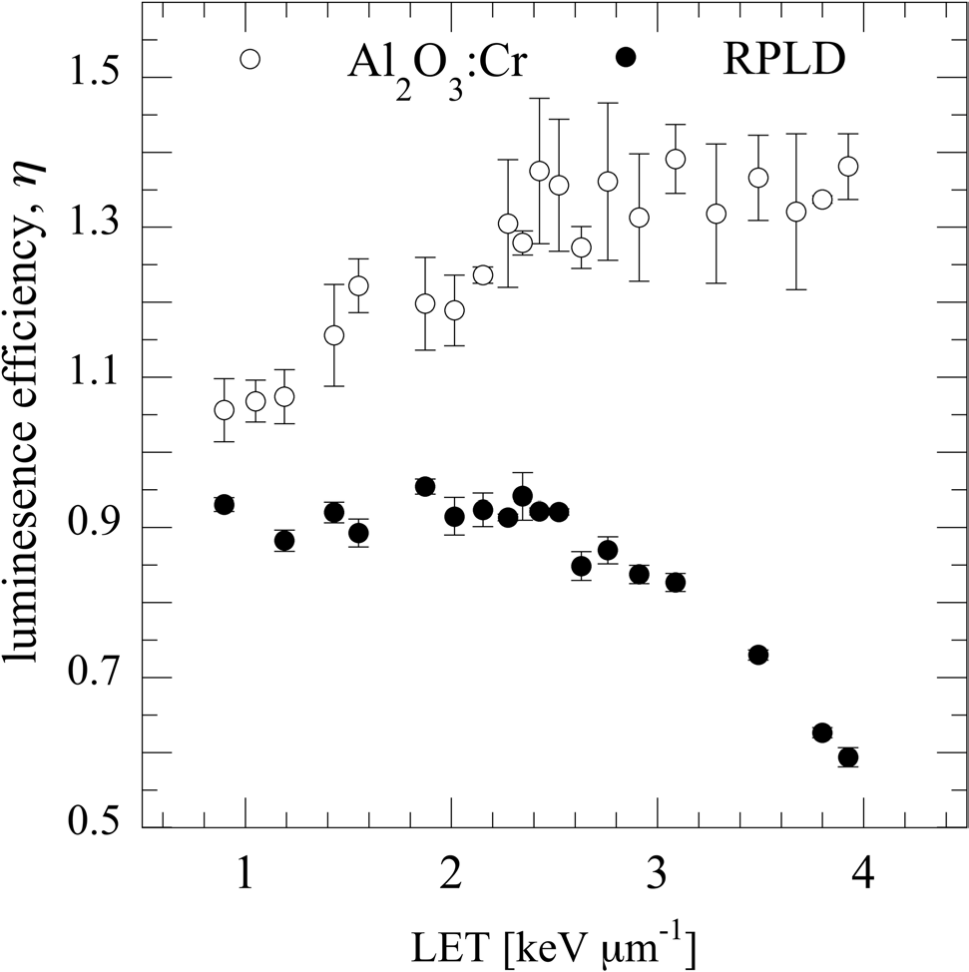
${\eta }_{\text{RPLD}}$ and ${\eta }_{{\text{Al}}_{2}{\text{O}}_{3}:\text{Cr}}$ as functions of LET

**Fig. 5:**
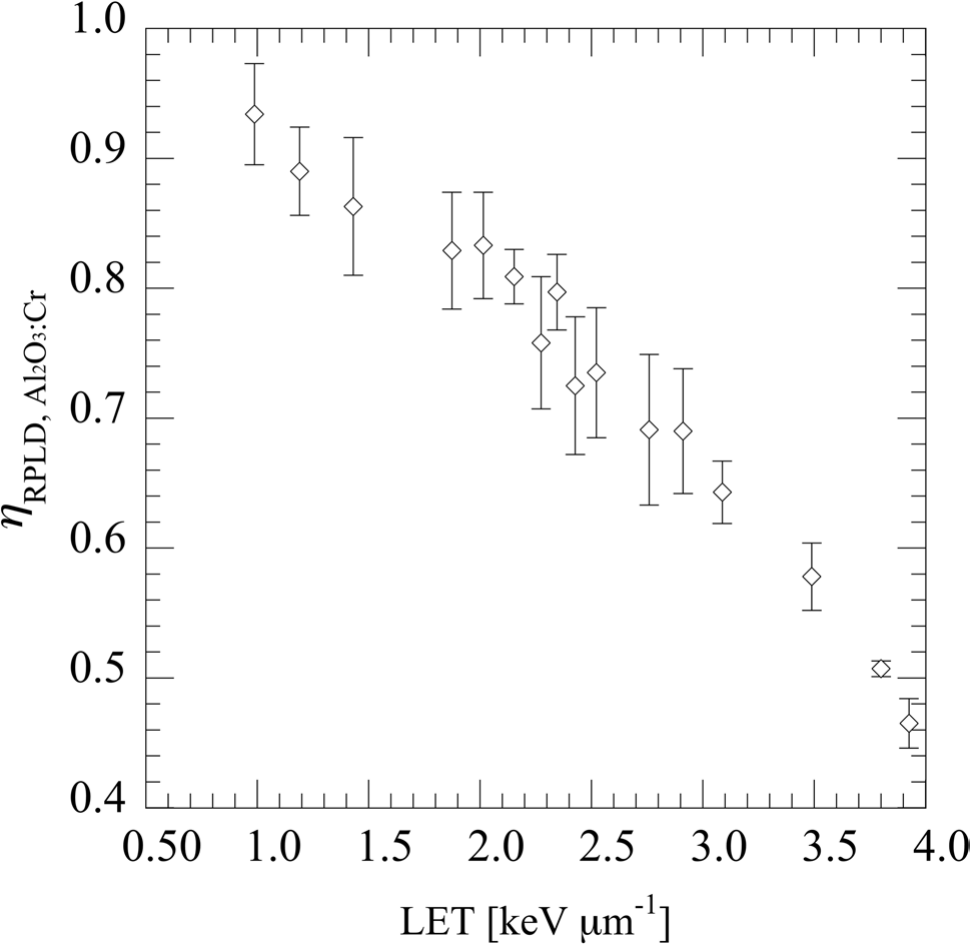
Relationship between ${\eta }_{{\text{RPLD}, \, \text{Al}}_{2}{\text{O}}_{3}:\text{Cr}}$ and LET

Figure 4 shows the luminescence efficiencies of the RPLD ${\eta }_{\text{RPLD}}$ and Al_2_O_3_:Cr TLD ${\eta }_{{\text{Al}}_{2}{\text{O}}_{3}:\text{Cr}}$ as functions of LET. ${\eta }_{\text{RPLD}}$ decreased with increasing LET, whereas ${\eta }_{{\text{Al}}_{2}{\text{O}}_{3}:\text{Cr}}$ increased with increasing LET. The error bars represent the standard deviation of the measured results. The standard deviation of the Al_2_O_3_:Cr TLD was relatively larger than RPLD, with the maximum standard deviation reaching 8.8%. Nevertheless, the average coefficients of variation for Al_2_O_3_:Cr TLD and RPLD were 4.5% and 1.7%, respectively. To clarify the relationship between ${\eta }_{\text{RPLD}}$ and ${\eta }_{{\text{Al}}_{2}{\text{O}}_{3}:\text{Cr}}$, the dose ratio of the RPLD and Al_2_O_3_:Cr TLD ${\eta }_{\text{RPLD}, {\text{Al}}_{2}{\text{O}}_{3}:\text{Cr}}$ are shown in Fig. 5. ${\eta }_{\text{RPLD}, {\text{Al}}_{2}{\text{O}}_{3}:\text{Cr}}$ decreased from 0.934 to 0.465 as the LET increased to 4 keV µm^−1^.

**Fig. 6:**
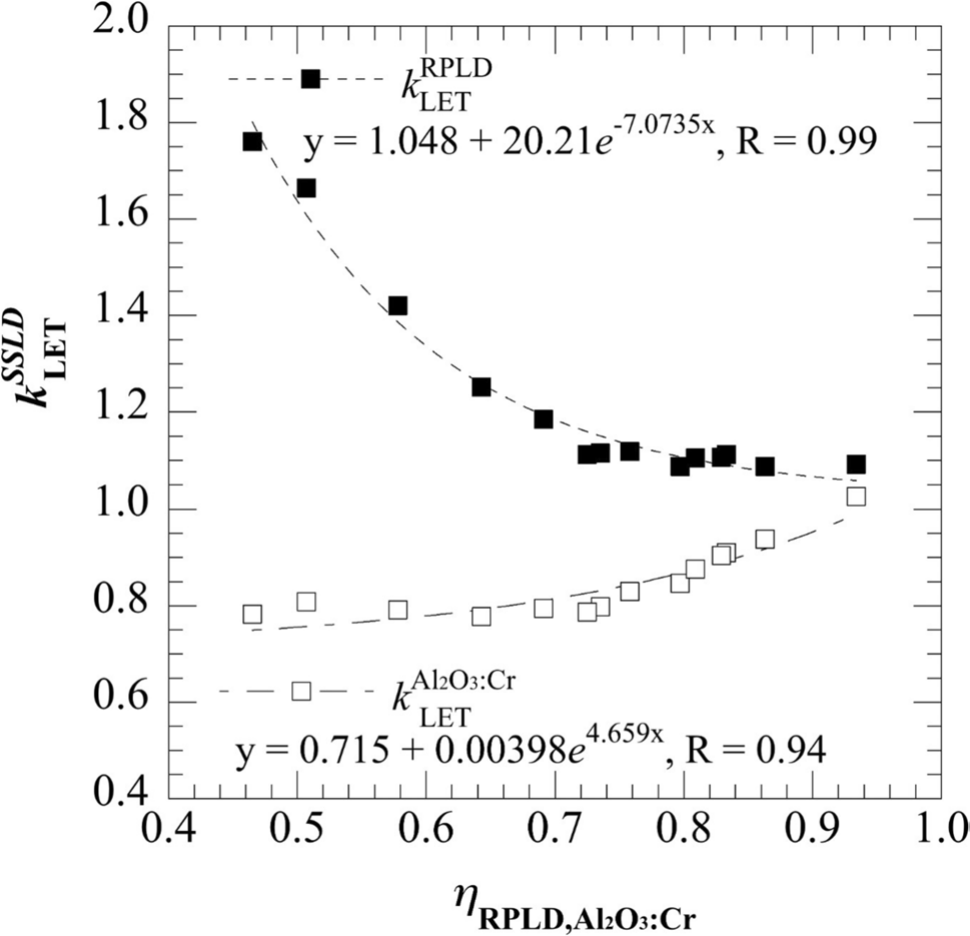
${\eta }_{\text{RPLD}, {\text{Al}}_{2}{\text{O}}_{3}:\text{Cr}}$ as a function of LET-dependent correction factor for the RPLD (${k}_{\text{LET}}^{\text{RPLD}}$) and the Al_2_O_3_:Cr TLD (${k}_{\text{LET}}^{{\text{Al}}_{2}{\text{O}}_{3}:\text{Cr}}$), respectively

LET can be eliminated by combining Figs. 4 and 5. The relationship between the $\eta$ and the ${\eta }_{\text{RPLD}, {\text{Al}}_{2}{\text{O}}_{3}:\text{Cr}}$ was obtained. Consequently, the LET correction factor, the reciprocal of the luminescence efficiency, for the RPLD (${k}_{\text{LET}}^{\text{RPLD}}$) and the Al_2_O_3_:Cr TLD (${k}_{\text{LET}}^{{\text{Al}}_{2}{\text{O}}_{3}:\text{Cr}}$) can be expressed by the equations shown in Fig. 6.

### SOBP beam

**Fig. 7:**
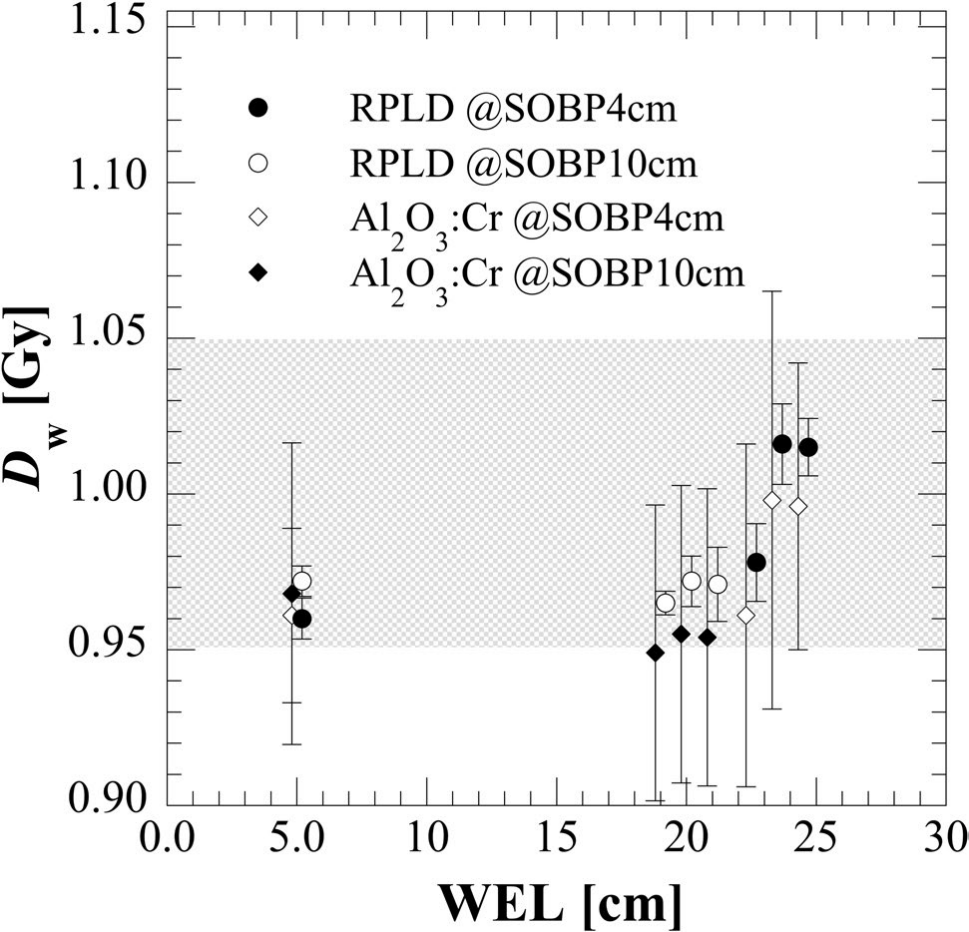
*D*_w_ obtained with RPLD and Al_2_O_3_:Cr TLD at different depths in the modulated (SOBP = 4, 10 cm) proton beams. Both dosimeters were set at the same WEL in the experiments. The artificial shifting of data points ensures clear visualization of overlapping measurements

Figure 7 shows *D*_w_ obtained using RPLD and Al_2_O_3_:Cr TLD according to Eq. (1) in the modulated (SOBP = 4, and 10 cm) proton beams. The gray-painted area indicates the criteria for postal dosimetry audit of ± 5% [21]. The error bars represent the standard deviation by the measurement of four SSLDs. Mean of the coefficient of the deviation for RPLD and Al_2_O_3_:Cr TLD were 0.87% and 3.74%, respectively. To correct the LET-dependent response, the correction factors shown in Fig. 6 were applied to correct the LET-dependent response. After LET dependence correction, the dose differences between ${D}_{w}^{\text{RPLD}}$ and ${D}_{w}^{\text{IC}}$ were between − 3.98% and 1.59% and those between ${D}_{w}^{{\text{Al}}_{2}{\text{O}}_{3}:\text{Cr}}$ and ${D}_{w}^{\text{IC}}$ were between − 5.11% and − 0.39%.

## Discussion

The LET-dependent efficiencies of the RPLD and Al_2_O_3_:Cr TLD were investigated in detail using an unmodulated 235 MeV proton beam and the feasibility of the two-dosimeter-methodology was validated using the modulated (SOBP = 4, and 10 cm) proton beams. Meanwhile, *D*_w_ estimated using RPLD and Al_2_O_3_:Cr TLD showed dose differences with averages of 1.77% and 2.35%, respectively.

**Fig. 8:**
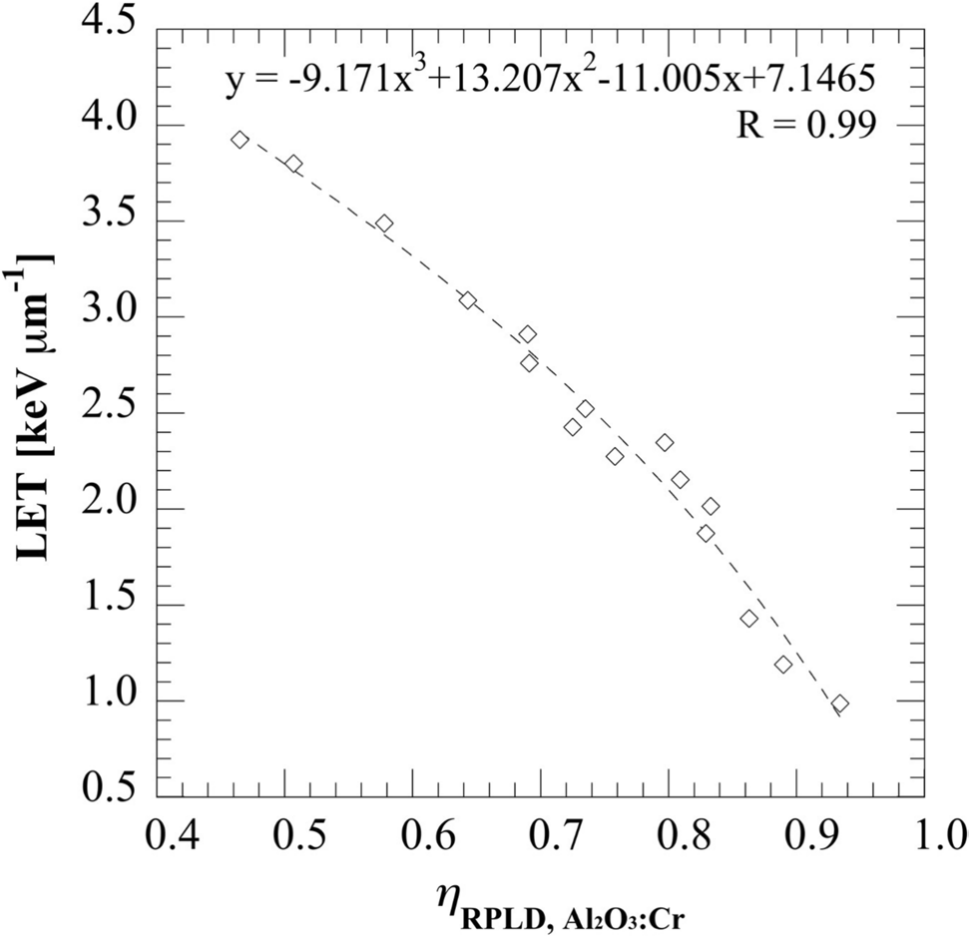
${\eta }_{\text{RPLD}, {\text{Al}}_{2}{\text{O}}_{3}:\text{Cr}}$ as a function of LET

To further analyze the reason for the underestimation of *D*_w_ using SSLDs with the proposed method, we re-plotted the LET as a function of ${\eta }_{{\text{RPLD}, \, \text{Al}}_{2}{\text{O}}_{3}:\text{Cr}}$ (Fig. 8) and estimated the LETs from ${\eta }_{{\text{RPLD}, \, \text{Al}}_{2}{\text{O}}_{3}:\text{Cr}}$. The ${\eta }_{{\text{RPLD}, \, \text{Al}}_{2}{\text{O}}_{3}:\text{Cr}}$ and the LET values at each measured depth are listed in Table 3. For both SOBP beams, the estimated LETs in the plateau region were smaller than those in the SOBP region. According to the previous study [22], the average LET is between ~ 2.0 keV μm^−1^ and ~ 3.0 keV μm^−1^ (decreasing with the depth position of the SOBP center) in the SOBP center for a 10 cm SOBP beam which was consistent with our results. Moreover, in the SOBP region, the estimated LETs for the 10 cm SOBP were all smaller than that for the 4 cm SOBP which is reasonable as the SOBP center for 4 cm SOBP was closer to the distal fall-off of the SOBP. Though this comparison alone does not constitute a full validation. In this study, independent Monte Carlo simulations for LET verification were not performed due to limited geometry information for the ridge filter used in the beam delivery system, and detailed published LET profiles matching our beam conditions are limited.

**Table 3:** LET estimated using ${\eta }_{\text{RPLD}, {\text{Al}}_{2}{\text{O}}_{3}:\text{Cr}}$

Location		Plateau	1 cm proximal to the SOBP center	SOBP center	1 cm distal to the SOBP center
4 cm SOBP	${\eta }_{\text{RPLD}, {\text{Al}}_{2}{\text{O}}_{3}:\text{Cr}}$	0.891	0.746	0.695	0.682
	LET [keV µm^−1^]	1.335	2.476	2.797	2.873
10 cm SOBP	${\eta }_{\text{RPLD}, {\text{Al}}_{2}{\text{O}}_{3}:\text{Cr}}$	0.876	0.785	0.768	0.762
	LET [keV µm^−1^]	1.473	2.209	2.331	2.371

The uncertainty in the proposed methodology, was mainly attributed to ${k}_{\text{LET}}^{\text{RPLD}}$ and ${k}_{\text{LET}}^{{\text{Al}}_{2}{\text{O}}_{3}:\text{Cr}}$. The uncertainties for the correction factors were combined including (1) the uncertainty of the experimental ${\eta }_{\text{RPLD}}$ and ${\eta }_{{\text{Al}}_{2}{\text{O}}_{3}:\text{Cr}}$, (2) the estimated LET, and (3) the regression curve for the ${k}_{\text{LET}}^{\text{RPLD}}$ and the ${k}_{\text{LET}}^{{\text{Al}}_{2}{\text{O}}_{3}:\text{Cr}}$ (Fig. 6). The uncertainties of the experimental ${\eta }_{\text{RPLD}}$ and ${\eta }_{{\text{Al}}_{2}{\text{O}}_{3}:\text{Cr}}$ were the combined uncertainty contributed by the type A uncertainty of ${M}^{\text{SSLD}}$ and ${D}_{w}^{\text{IC}}$ which were estimated as 0.99% and 2.3% for ${\eta }_{\text{RPLD}}$ and ${\eta }_{{\text{Al}}_{2}{\text{O}}_{3}:\text{Cr}}$, respectively. The uncertainty of the estimated LET was 1.35% from a previous study [8]. The uncertainty from the regression curves for the ${k}_{\text{LET}}^{\text{RPLD}}$ and the ${k}_{\text{LET}}^{{\text{Al}}_{2}{\text{O}}_{3}:\text{Cr}}$ was classified as type B with a rectangular distribution following the approach by Mizuno et al. [23]. The values were estimated to be 1.7% and 5.2%, respectively, based on the observed maximum deviation between the experimental points and the fitting curve. The combined uncertainties for the correction factors of the RPLD and the Al_2_O_3_:Cr TLD were 2.39% and 5.84%, respectively. The uncertainty of our proposed methodology was relatively large for the postal dosimetry audit criteria. A major contributor to the uncertainty of the Al₂O₃:Cr TLD is from ${M}^{\text{SSLD}}$. Previously Maruyama et al. estimated the uncertainty of the Al₂O₃:Cr TLD as 1.8% [11], which is significantly lower than the uncertainty estimated in our protocol. This discrepancy is largely attributed to differences in readout system performance. We are currently developing a new readout system aimed at improving reproducibility and reducing statistical noise. Even though the uncertainty may be relatively large, the purpose of this study was to show the protentional of the two-dosimeter LET correction methodology and highlights the potential for improvement with refined instrumentation. In addition, a slight discrepancy on the ${\eta }_{\text{RPLD}}$ between this study and previous studies [8–10] should be noted. ${\eta }_{\text{RPLD}}$ decrease by around 10% to 15% at LET = 1–3 keV µm^−1^ in previous studies [8–10], while it was found a gradual decrease in ${\eta }_{\text{RPLD}}$ in this LET region. In Nagata et al., the overall variation in ${\eta }_{\text{RPLD}}$ was within 1% in LET range of 1.4–2.8 keV µm^−1^ [10]. Moreover, in the study by Chang et al., ${\eta }_{\text{RPLD}}$ was reported to decrease significantly from approximately 0.88 to 0.70 as LET increased from 1 to 3 keV µm^−1^ indicating a sharper decline compared with our results. Nevertheless, at higher LET values around 4 keV µm^−1^, our findings are consistent with Chang et al.’s results, where ${\eta }_{\text{RPLD}}$ decreased to approximately 0.6. Possible reasons for the discrepancies include the differences in beam conditions, and readout procedures [8]. As we followed Chang et al.’s readout procedure that read out within 1 week after irradiation following a consistent preheating protocol, and individually calibrated using a 6-MV photon beam, maybe the difference results by the RPLD batch properties.

Another limitation of this study is that the validation experiment was only performed on a passive proton beam delivery system. Since the scanning beam delivery systems are being widely adopted, the feasibility of the proposed methodology for scanning proton beams needs to be investigated. Despite these limitations, the concept of combining two detectors with complementary LET-dependent responses presents a significant advantage—to estimate LET and derive LET-dependent correction factors directly, without relying on external beam quality indices such as the residual range or pre-calculated LET. Although the accuracy of the present method remains limited, the proposed two-dosimeter approach demonstrates strong potential to simplify LET correction procedures and enhance the practicality of proton dosimetry.

## Conclusion

In this study, we developed a two-dosimeter methodology for deriving the LET dependence correction factor and validated it using therapeutic SOBP proton beams. Proton dosimetry based on the proposed methodology underestimated the *D*_w_ by an average of 1.88% and 3.21% for the RPLD and Al_2_O_3_:Cr TLD, respectively. This demonstrated the feasibility of the proposed methodology. By employing two SSLDs with opposing LET-dependent responses, the need for ionization chambers is eliminated, which is advantageous for postal dosimetry applications. Although the current uncertainty levels are relatively high, further refinement of readout systems, which is currently underway, is expected to improve the accuracy and precision. Therefore, we concluded that the two-dosimeter methodology is promising for LET correction.

## Data Availability

The data that support the finding of this study are available from the corresponding author on reasonable request.
